# Using cognitive interviews to improve a measure of organizational readiness for implementation

**DOI:** 10.1186/s12913-022-09005-y

**Published:** 2023-01-27

**Authors:** Maria McClam, Lauren Workman, Emanuelle M. Dias, Timothy J. Walker, Heather M. Brandt, Derek W. Craig, Robert Gibson, Andrea Lamont, Bryan J. Weiner, Abraham Wandersman, Maria E. Fernandez

**Affiliations:** 1 Center for Applied Research and Evaluation, Arnold School of Public Health, University of South Carolina, Columbia, SC USA; 2 Department of Health Services, Policy, and Management, Arnold School of Public Health, University of South Carolina, Columbia, SC USA; 3 The University of Texas Health Science Center at Houston School of Public Health, Houston, TX USA; 4 St. Jude Children’s Research Hospital, Memphis, TN USA; 5 Augusta University, Augusta, GA USA; 6 Wandersman Center, Columbia, SC USA; 7 Department of Global Health, University of Washington, Seattle, WA USA

**Keywords:** Organizational readiness, Qualitative methods, Cognitive interviewing, Evidence-based interventions, cancer prevention, Measure development

## Abstract

**Background:**

Organizational readiness is a key factor for successful implementation of evidence-based interventions (EBIs), but a valid and reliable measure to assess readiness across contexts and settings is needed. The R = MC^2^ heuristic posits that organizational readiness stems from an organization’s **m**otivation, **c**apacity to implement a specific innovation, and its general **c**apacity. This paper describes a process used to examine the face and content validity of items in a readiness survey developed to assess organizational readiness (based on R = MC^2^) among federally qualified health centers (FQHC) implementing colorectal cancer screening (CRCS) EBIs.

**Methods:**

We conducted 20 cognitive interviews with FQHC staff (clinical and non-clinical) in South Carolina and Texas. Participants were provided a subset of items from the readiness survey to review. A semi-structured interview guide was developed to elicit feedback from participants using “think aloud” and probing techniques. Participants were recruited using a purposive sampling approach and interviews were conducted virtually using Zoom and WebEx. Participants were asked 1) about the relevancy of items, 2) how they interpreted the meaning of items or specific terms, 3) to identify items that were difficult to understand, and 4) how items could be improved. Interviews were transcribed verbatim and coded in ATLAS.ti. Findings were used to revise the readiness survey.

**Results:**

Key recommendations included reducing the survey length and removing redundant or difficult to understand items. Additionally, participants recommended using consistent terms throughout (e.g., other units/teams vs. departments) the survey and changing pronouns (e.g., people, we) to be more specific (e.g., leadership, staff). Moreover, participants recommended specifying ambiguous terms (e.g., define what “better” means).

**Conclusion:**

Use of cognitive interviews allowed for an engaged process to refine an existing measure of readiness. The improved and finalized readiness survey can be used to support and improve implementation of CRCS EBIs in the clinic setting and thus reduce the cancer burden and cancer-related health disparities.

**Supplementary Information:**

The online version contains supplementary material available at 10.1186/s12913-022-09005-y.

## Contributions to the literature

- This study helps to advance the field of implementation science by developing a valid and reliable measure that aligns with the R = MC2 heuristic to increase implementation success.
- This study provides an example of how to use cognitive interviewing to improve measurement tools.
- This study confirms some of the common pitfalls known in survey design and measurement tools.
- This paper demonstrates how an improved readiness survey can contribute to implementation of evidence-based interventions for cancer prevention and control.

## Background

Colorectal cancer (CRC) is the second most common cause of cancer death in the United States (US) [1, 2]. The US Preventive Services Task Force recommends CRC screening (CRCS) for adults aged 45-75 who are at average risk [3]. While CRCS rates have increased in the years prior to the COVID-19 pandemic, they still lag behind national goals and the pandemic caused additional delays or halts in screening [4]. For example, recent estimates suggest 65.2% of adults were screened, while the target for US Department of Health and Human Services (DHHS) *Healthy People 2030* goal is 74.4% [5] and the National Colorectal Cancer Roundtable’s goal is to achieve 80% CRCS rates in every community [6]. Moreover, CRCS rates are disproportionate in racial and ethnic groups, and disparities in screening uptake persist [7]. For example, CRCS uptake is highest among Whites and lowest among Hispanics [8].

Federally qualified health centers (FQHCs) provide affordable healthcare for many Americans, many of which are at or below the federal poverty level and come from underserved communities with lower CRCS rates [9]. Despite serving many patients, CRCS rates among FQHCs (40.1% in 2020) remain below national averages (65.2%) [10, 11]. CRCS is also a Uniform Data System clinical quality measure for health centers. To help increase CRCS rates, FQHCs utilize evidence-based interventions (EBIs), such as provider assessment and feedback, provider reminders, client reminders, and reducing structural barriers [12, 13]. .EBIs provide guidance on strategies to implement and promote use of CRCS [14]. Additionally, the Guide to Community Preventive Services (the Community Guide) [15] disseminates recommended EBIs. Despite having these EBIs available, implementation remains a challenge; Hannon et al. and Adams et al. found FQHCs often discontinue an EBI because of capacity issues [16, 17]. Thus, there is a gap in the motivation and capacity to effectively implement and sustain EBIs to improve CRCS. For example, when electronic health records cannot support integration of provider reminder systems or provider assessment and feedback reports, uptake, implementation success and the sustainability of the EBI is compromised. Additionally, provider related EBIs require strategic partnerships that take time to build, showing readiness can be an ongoing and shifting process [17]. Moreover, CRCS is often a lower priority for providers, especially amongst patients with multiple chronic conditions or complex medical histories [18]. Several initiatives exist to increase implementation of EBIs to promote CRCS in FQHCs including the Centers for Disease Control and Prevention’s (CDC) Colorectal Cancer Control Program [19], the Cancer Prevention and Control Research Network (CPCRN) [16], the American Cancer Society’s (ACS) Community Health Advocates Implementing Nationwide Grants for Empowerment and Equity (CHANGE) grant program [20], and the Evidence-Based Cancer Control Programs (EBCCP) [21].

In the health care setting, understanding and attending to organizational level barriers and organizational readiness has been associated with implementation success [22–24]. Readiness represents a central construct in several implementation science frameworks including the Interactive Systems Framework for Dissemination and Implementation (ISF) [25], the Consolidated Framework for Implementation Research (CFIR) [26], Getting To Outcomes [27], and Context and Capabilities for Integrating Care [28]. Organizational readiness plays a role during all phases of program implementation [22] and reflects the organizations’ commitment, motivation, and capacity for change over time [24]. This idea of readiness emerged from the ISF [22, 25]. Informed by the ISF [22, 25] and past research identifying the importance of organizational capacity [29] and motivation [23, 30], Scaccia et al. [22] developed a heuristic for organizational readiness known as R = MC^2^. The R = MC^2^ heuristic proposes that readiness is made up of three distinct components: the organization’s *motivation* to implement an innovation, *general organizational capacities*, and *innovation specific capacities*.

Organizational readiness is critical to successful implementation, yet there is a need for a valid and reliable measure that aligns with the R = MC2 heuristic to increase implementation success [22, 23, 31–34]. A readiness survey was originally developed based on the R = MC^2^ framework to assess and monitor readiness for implementing a health improvement process among community coalitions and has since been used in other settings [35, 36]. For example, one study applied the readiness survey in a mixed methods approach among primary care and specialty clinics, pharmacies within health systems, and community pharmacies. They found engaging in the readiness work was associated with many benefits including increased awareness of readiness challenges, ensuring alignment of priorities, and making sure the intervention was a good fit [37]. Another study adapted the readiness survey to assess organizational readiness for integrated care and developed a Readiness for Integrated Care Questionnaire (RICQ). The tool was then piloted with 11 health care practices that serve vulnerable, underprivileged populations [36]. The readiness survey has further been applied to operationalize readiness building in a variety of settings. Using the readiness survey is the first of three stages (assessment, feedback and prioritization, strategize) to develop and test practical strategies for supporting implementation in real-world settings [38]. While used before, the readiness survey had not been rigorously evaluated in terms of its psychometric properties or used in FQHC settings to assess readiness for implementation of cancer control interventions. This study represents part of a rigorous process of adaptation, validation and testing of the readiness survey which is ultimately intended to be used in multiple settings for a variety of implementation efforts.

To be a well-established measure, the readiness survey must demonstrate adequate levels of reliability and validity [39]. The measure development process can include initial item review from respondents and obtaining feedback to improve measures; however, there are few examples in the research literature of having members of the intended response community review items for interpretability and clarity. Cognitive interviewing is a widely used method used to improve understanding of question validity and reduce response error [40]. Cognitive interviewing (sometimes called learner verification) is a process by which participants verbalize their thought processes while responding to written text, such as a survey. Cognitive interviews may be used to examine the clarity of meaning for words and phrases, the cognitive process used for arriving at an answer, identify problems with the measure’s instructions, and the optimal order and context for information as presented to the interviewee [41, 42].

The work described in this article was part of a larger study to further develop, refine and test the previously developed readiness survey [24]. The larger study consists of multiple phases that include both qualitative and quantitative analyses [24]. Results presented in this paper represented one of the qualitative phases of this larger, more lengthy measure development process [43]. The overall goal of the larger study is to adapt, further develop, and evaluate the validity and reliability of the existing readiness survey so that it can be used across settings and topic areas to assess readiness to inform implementation strategy development or other efforts to improve implementation of evidence-based interventions. The purpose of this paper was to describe a qualitative process used to assist improvement of the existing measure of readiness.

## Methods

This study used a narrative research approach to guide our qualitative work. The narrative research approach helped us understand individuals’ experiences with the readiness survey [44]. The research team that completed this study is diverse with a range of qualifications, experiences, and familiarity with FQHCs. This allowed for the integration of diverse perspectives in the research design, analysis, and interpretation of findings. We used the Standards for Reporting Qualitative Research (SRQR) checklist when compiling this manuscript. The Committee for Protection of Human Subjects at the institutions associated to this study reviewed and approved all procedures and protocols.

### Setting and recruitment

We recruited participants from 11 FQHC systems in South Carolina (SC) and Texas (TX). We used a purposive sampling approach to recruit participants [45] by leveraging existing relationships with FQHCs. In TX, we worked with a clinic contact (e.g., nurse manager) to provide us with a list of possible participants and accompanying email addresses for us to reach out to for interview participation. In SC, we directly emailed FQHC contacts that the research team had previous relationships with from other projects related to implementing CRCS initiatives. We collected data between December 2020 and February 2021. All cognitive interviews were conducted using a virtual online video platform (Zoom or WebEx) and were approximately one-hour in length. Interviews were conducted virtually because of the COVID-19 pandemic. Interviews were audio-recorded, and professionally transcribed verbatim. Interview participants were compensated with $75 e-gift cards for their time.

### Interviews

This study used a qualitative, semi-structured cognitive interview approach to gather feedback on the existing items included on the readiness survey. The research team developed a cognitive interview guide that included a general debriefing discussion about first impressions of the readiness survey and specific questions about each item consistent with the cognitive interview methods described by Beatty and Willis [41, 46]. The readiness survey was shared with participants prior to the interview.

Three research team members (DC, ED, MM) trained in cognitive interviewing techniques completed the interviews. We asked interview participants to think aloud or describe their thought process out loud as they answered questions. Then, we went through items on the readiness survey one at a time with the participant. Given the length of the readiness survey and because the interviews were being conducted virtually, the research team divided the readiness survey into subsets and participants were asked about only a portion of the readiness survey; thus, keeping the interviews to approximately 1 h long.

Interviews focused on the following topics: 1) what the participant was thinking about when they read a question, 2) how easy/difficult questions were to answer, 3) how the questions could be improved, and 4) how they interpreted specific terms in the questions. Table 1 describes the purpose and examples of interview questions. We asked participants a series of questions regarding the readiness survey instructions including: “*What are your general impressions of the Readiness Survey instructions*?” “*What in the instructions did you think was unclear?”* and *“How would you improve this*?” Data were collected from at least two interview participants per item on the readiness survey. In addition, we administered a brief descriptive questionnaire before the interview to ascertain sociodemographic information from participants. The reporting of our qualitative study is guided by the Standards for Reporting Qualitative Research (SRQR) checklist [47].

**Table 1:** Purpose and examples of cognitive interview questions

Purpose of Interview Question	Example of Interview Questions
Identify cognitive processes used for answering items. Determine if similar items are perceived as being redundant.	*Tell me what you were thinking as you read this question*
Identify if items are confusing or straight forward.	*How easy/difficult did you find this question to answer? Why do you say that?*
Determine suggestions for wording changes or re-phrasing a question.	*How could the question be improved?*
Explore the meaning of specific words or phrases and determine if items are culturally appropriate.	*What does the term ____ mean to you?*

### Data analysis

We used an inductive approach to analyze transcripts that allows themes to emerge from data [48]. We coded and analyzed the transcripts using a qualitative analysis software (ATLAS.ti 9 Windows). Two researchers (ED, MM) used open coding to review interview data independently. Both researchers then discussed coded transcripts to share any new codes and develop a consensus. A working codebook was developed and updated as new codes emerged. We reviewed the data until saturation was reached (no new themes or ideas emerged from the data) and recurring themes were identified [49].

We then mapped the transcripts back to each survey item in the readiness survey. A summary of the participant’s recommended changes was constructed for each survey item and organized in Microsoft Excel. The Excel document’s column headings included: Current Readiness survey Item, Participant Response (Interview 1), Participant Response (Interview 2), Summary of Participant’s Recommendations, Analysis Team’s Thoughts, Refinement Level (minor, major, no change), Proposed New Item, What Change Was Made (brief explanation of what the change was) (Additional file 1). Our comprehensive Excel document was used for summarizing changes to all subsets of the readiness survey, including the two interviews per subset of items.

This Excel document was then shared with the rest of our analysis team, which consisted of three more team members (LW, AL, TW). Our analysis team went through interview data in the Excel document and discussed each readiness survey item. Proposed changes for any items were also recorded in the Excel document. Proposed new items were color coded for major and minor edits. Finally, a third expert review team consisting of three readiness survey experts (MEF, BW, AW) reviewed suggestions and proposed final modifications to the readiness survey items based on the interview data. All steps leading up to the proposed change were recorded in a table (Additional file 2) that included: Current Readiness Survey Item, Interview Responses, Summary of Participants’ Recommendation, Analysis Team Thoughts, and Proposed New Readiness Survey Item.

## Results

### Participants

A total of 20 individuals from FQHC clinics across SC (*n* = 9) and TX (*n* = 11) participated in the cognitive interviews. Participants represented 11 different FQHC systems across SC and TX, in addition to the SC Primary Health Care Association (SCPHCA). The SCPHCA was included because of their close relationships with FQHC staff and their unique perspective as the unifying organization for SC FQHCs. Participants represented a variety of roles or job types (e.g., quality improvement directors, nurses, medical assistants). This allowed for multiple perspectives given a variety of staff members representing different roles would be taking the survey. Participants were mostly female (75%), Black/African American (40%), and middle aged (35-44, 45%). Characteristics of all interview participants are shown in Table 2.

**Table 2:** Characteristics of cognitive interview participants (*n* = 20)

	***f*** (%)
Age	
30-34	3 (15%)
35-39	4 (20%)
40-44	5 (25%)
45-49	2 (10%)
50-54	4 (20%)
55-59	2 (10%)
Sex	
Male	5 (25%)
Female	15 (75%)
Race/Ethnicity	
White, non-Hispanic	6 (30%)
White, Hispanic	5 (25%)
Black/African American, non-Hispanic	8 (40%)
Asian	1 (5%)
Native Language	
English	17 (85%)
Spanish	2 (10%)
Other	1 (5%)
How many years have you worked at this clinic?	
0-2 years	4 (20%)
3-5 years	9 (45%)
6 or more years	7 (35%)
Role	
Quality Improvement Director/Manager	6 (30%)
Nurse Practitioner/Physician Assistant/Nurse	3 (15%)
Medical Assistant/Clinical Assistant/Medical Manager	5 (25%)
Other Leadership (CFO, Clinical Director)	2 (10%)
Other (IT, Front Desk, Grants Coordinator)	4 (20%)

### Overall readiness survey feedback

Interview participants provided constructive feedback and suggested recommendations to improve the readiness survey:

- *The Survey “Needs to be Shortened.”* Overall, participants felt that the readiness survey was too long and needed to be shortened. A participant explained how long surveys are less likely to be completed: “*The shorter a survey is, the more likely the people will complete it.*” One participant explained how she will close out of surveys if they are too long: *“When you send a survey…and I get them all the time, I usually click on it. I try to gauge on the first page how long the survey by the percentage. But when I answer the first couple of questions, If I’m at 8% completed, I’m going to close... I don’t have time to sit there for 30 minutes and answer your 40 questions. If I’m 20 or 25%, which tells me it’s only four or five questions, I’m more likely to finish it.”*
- *The Introduction is “Clear and Concise.”* Participants expressed that the introduction language for the survey was clear. A participant explained that *“it’s kind of just standard evaluation language and nothing that stood out as confusing.”*
- *A 7-point Likert Scale is Challenging*. One participant suggested that using a 7-point Likert scale would be more challenging to answer than a 5-point Likert scale. She described, “*I can see after a while people may be get inconsistent with, do I pick a two or three... I’ve not done that much detail in a Likert scale, so that may be a little bit of a concern.*”

### Item redundancy

A key recommendation that participants had was to remove items they interpreted to be duplicative. For example, multiple participants stated that they felt that the following three items were too similar: “people can safely tell their coworkers about any mistakes they make,” “people feel comfortable telling their coworkers about any mistakes they make,” and “people feel it is safe to admit any mistakes they make to a coworker”. Participants went further to explain that they liked “people feel comfortable telling their coworkers about any mistakes they make” the best out of the three items because it most straight forward.

### Clarifying terminology

#### Intervention vs. innovation

Participants noted the importance of using terminology that is common in the clinic setting. For example, in their clinics, they are more familiar with the term intervention instead of innovation (as used in the readiness survey). Intervention is used in the well-known term “evidence-based intervention” (EBI). Participants recommended switching out innovation for intervention throughout the entire survey. For example, one participant explained, “*When I think of innovation, to me, innovations is something that you’re leading in, almost like something you designed... and they haven’t necessarily designed this… innovation isn’t a word that I think that I would use.”*

#### Pronouns and vague terminology

An additional recommendation included clarifying vague items. For example, one item “we communicate well with each other” was interpreted as vague and open for interpretation. Participants recommended clarifying who “we” is. For example, “we feel confident in our ability to implement this innovation,” confused participants on whether the research team was referring to a particular clinic, staff, or the entire FQHC system. Vague words were defined by participants throughout the cognitive interviews. Participants discussed that using certain terms like “our organization”, “people”, and “we” could mean different things in different items. For example, “our organization” and “people” were both defined by participants as meaning leadership/board members or staff. Participants recommended clarifying these vague terms to define either leadership or all clinic staff. In another example, the item “our clinic is among the first to try new ways of doing things” was also interpreted as being vague. A participant explained how “things” is a vague term: “*community outreach things? I think that “things“ would need to defined*.” Additionally, some participants recommended specifying ambiguous terms like “better.” When reading the following items: 1) “this innovation is better than other innovations we have used before in our clinic” and 2) “the innovation meets our clinic needs better than what we have been doing,” several participants asked: “how is it better than before?” and “what do you mean by better?” In the item “the people in our clinic value others’ unique skills and talents,” participants indicated that they weren’t sure what “others” meant. They suggested this term could mean other organizations or other colleagues.

#### Words with more than one meaning

Participants also highlighted terms that could have more than one meaning. Additional terms for clarification noted by participants included “minority,” “take the time,” and “others.” Minority was defined by participants as “*people not in leadership”* or *“the minority among job titles in the clinical area.”* However, one participant noted that this could also be defined by race/ethnicity and suggested clarifying. Participants also recommended defining “take the time” because this could mean different things to different people. For example, one participant explained how people’s interpretations could differ: “*some people sat around to discuss how it worked, but I would think that that would be, you know, to run a report to see how it worked and then look at the- look at the data.*”

#### Phrases that are difficult to understand

In general, participants felt most of the items on the readiness survey were easy to understand. However, participants recommended removing the items that were difficult to understand like the item “people are not afraid to be themselves at work.” A participant explained, “*I don’t know how to interpret that, I know I’m not afraid to be myself at work… every new institution has their policies and procedures and work ethic, you know, but I don’t know if, they’re showing themselves in work, Um, it’s, a little bit in a difficult, or in a to understand, ideally for me.”*

### Changes made to the readiness survey

Examples of item changes are illustrated in Table 3. For example, participants felt the item “people in this clinic generally reflect on how things are going” used a vague term (“reflect”). Therefore, the research team changed the item to “people in this clinic generally talk about how things are going.” This wording was suggested by an interview participant. A further example is for the item “our clinic is innovative.” A participant explained how different roles and job types at the clinic might not know what we mean by innovative. Therefore, the review team decided to switch the item to say, “our clinic is open to changes in the way we do things.” A final example is changes made to the item “intra-organizational relationships are important for implementation.” Participants explained that intra-organizational was a confusing term and “*ambiguous.”* Therefore, after discussion, the review team decided it was important to clarify what is meant by intra-organizational relationships and to change the item to “relationships between staff members across departments are important for implementing this innovation.”

**Table 3:** Examples of item changes

Original Item	Modified Item
**Motivation**	
This innovation fits well with our clinic’s culture.	The innovation fits well with our clinic’s norms.
We are able to try out pieces or parts of this innovation.	We are able to try out parts or phases of this innovation.
In the near future, we expect to see the benefits of this innovation.	Within the next 90 days, we expect to see the benefits of this innovation.
**Innovation-Specific Capacity**	
We know the core components of this innovation.	We know the main parts of this innovation.
Supervisors monitor how this innovation is implemented.	Supervisors track how this innovation is implemented.
We can communicate well with other teams within our clinic about this innovation.	Staff from different departments communicate well with each other about this innovation.
Intra-organizational relationships are important for implementation.	Relationships between staff members across departments are important for implementing this innovation.
**General Capacity**	
Our rules and regulation allow for creativity.	Our policies and procedures allow for creativity.
People in this clinic generally *reflect* on how things are going.	People in this clinic generally *talk* about how things are going.
Our clinic is innovative.	Our clinic is open to changes in the way we do things.

## Discussion

Readiness assessments can be used to support and improve implementation of EBIs for cancer prevention and control and thus improve CRC outcomes. This paper described a process for collecting user-focused data to improve a comprehensive readiness measure based on the R = MC2 heuristic and assessed item understanding, its relevance to the healthcare setting/context, and general interpretations of the structure. A better measure of organizational readiness is an essential step towards informing strategies to improve implementation. This paper also provides an example of a rapid process to engage the intended response community in improving measurement tools. Despite challenges related to conducting this study during the COVID-19 pandemic, this study was able to gather opinions from a diverse range of voices (including job types), which strengthens the readiness survey. It is critical to ensure that tools are tailored to and representative of the intended audience.

Although the use of qualitative methods in implementation science is well established, there are not many published studies that describe use of cognitive interviewing for the development and refining of measures to assess contextual factors influencing implementation. This research provides opportunity to better understand the complexity of the implementation context, as well as incorporate a diverse range of perspectives to improve our measure of readiness [50, 51]. Qualitative approaches explore the complexity of human behavior (feelings, perceptions, experiences, and thoughts) and generate deeper understanding participants’ experiences in certain settings. Incorporating qualitative data into this study helps better apply the readiness survey to the intended setting it is designed for [52, 53]. Furthermore, using a comprehensive Excel document for summarizing changes to all subsets of the readiness survey was a good strategy because it helped organize a vast amount of information into an easily accessible format that multiple team members could use to make decisions on refining the readiness survey.

Within measure development approaches, there are common issues to avoid when developing items [54–57]. Our interview participants identified some key examples within our measure (i.e. avoiding jargon, avoiding vague terms, avoiding words that can mean the same thing). It can be difficult to identify these issues if we only rely on the measure developers or “expert” reviewers. Thus, our study adds an important example of advantages for this user testing stage in the measure development process. Our study also demonstrates how we processed the information so others can follow this approach.

A potential limitation of our study is that interviews were conducted using online conferencing platforms (e.g., Zoom, WebEx) instead of in person. The ideal format for cognitive interviews is in person so the interviewer can see and observe body language [58]. However, we were unable to do the interviews in person because of the COVID-19 pandemic and this interviewing format facilitated reaching more participants during a critical time for FQHC clinics balancing many responsibilities in both South Carolina and Texas. A second limitation of our study was that we were only able to show interview participants a subset of items. We broke up the readiness survey into subsets because we wanted the virtual interviews to not last longer than 1 h. A third limitation of our study was that we had three participants who identified as non-native English speakers. This may have influenced the way in which they interpreted and/or responded to the items. A fourth limitation was that data were collected from only two to three interview participants per item on the readiness survey. Because we wanted feedback on a large number of items, we focused on breaking up the item sets for participants, so the interviews were more manageable. Recruitment of participants was also a challenge due to COVID-19 overwhelming health centers at the time of the interviews. Therefore, each item was only reviewed by two to three participants.

There is a need for a comprehensive measure of readiness. Overall, the goal of this study was to improve the readiness survey based on the R = MC^2^ framework (a measurement tool for readiness). Readiness is a critical step for successful implementation. This paper describes the use of cognitive interviews as part of a larger study [43] to validate the readiness survey, the next phase (developmental phase) of our study includes distributing the readiness survey to a large sample of FQHC clinics across the U.S. for continued testing and development. The cognitive interview data will be combined with quantitative data collected from FQHC clinics who completed the readiness survey. These data will be analyzed and integrated with the cognitive interviews to develop a final version of the readiness survey. From there, the readiness survey will be distributed again to a larger, national set of FQHC clinics for survey validation (validation phase). This novel mixed methods approach allows for a comprehensive development and validation of a measurement tool.

## Conclusion

Key recommendations included removing items interpreted as asking about the same concept and items that were difficult to understand. Additionally, participants recommended keeping terms consistent throughout the survey and changing pronouns (e.g., people, we) to be more specific (e.g., leadership). Moreover, participants recommended specifying ambiguous terms (e.g., define what “better” means).

By improving the readiness survey, the goal is to develop a theoretically-informed, pragmatic, reliable and valid measure of organizational readiness that can be used across settings and topic areas, by researchers and practitioners alike, to increase and enhance implementation of cancer control interventions. The finalized readiness survey will be used to support and improve implementation of EBIs for cancer prevention and thus reduce the cancer burden and cancer-related health disparities.

## Supplementary Information

**Additional file 1.** A Snapshot Example of the Excel Document Used for Data Analysis.

**Additional file 2.** A Snapshot Example of the Full Table Used to Keep Track of Steps Leading to Changes in the Readiness Survey.

## Data Availability

The interview data generated and analyzed during the current study are not publicly available to protect the privacy of those interviewed. However, the de-identified summarized tables represented in Additional files 1 and 2 may be available from the corresponding author on reasonable request.

## Abbreviations

EBIs: Evidence-based interventions
FQHC: Federally qualified health centers
CRCS: Colorectal cancer screening
ISF: Interactive Systems Framework for Dissemination and Implementation
SC: South Carolina
TX: Texas
