# Supplementary figures and images for: Using cognitive interviews to improve a measure of organizational readiness for implementation
Source: BMC Health Serv Res. 2023 Jan 27;23:93. doi: 10.1186/s12913-022-09005-y (PMC9881511; doi:10.1186/s12913-022-09005-y)

Additional file 1 A Snapshot Example of the Excel Document Used for Data Analysis


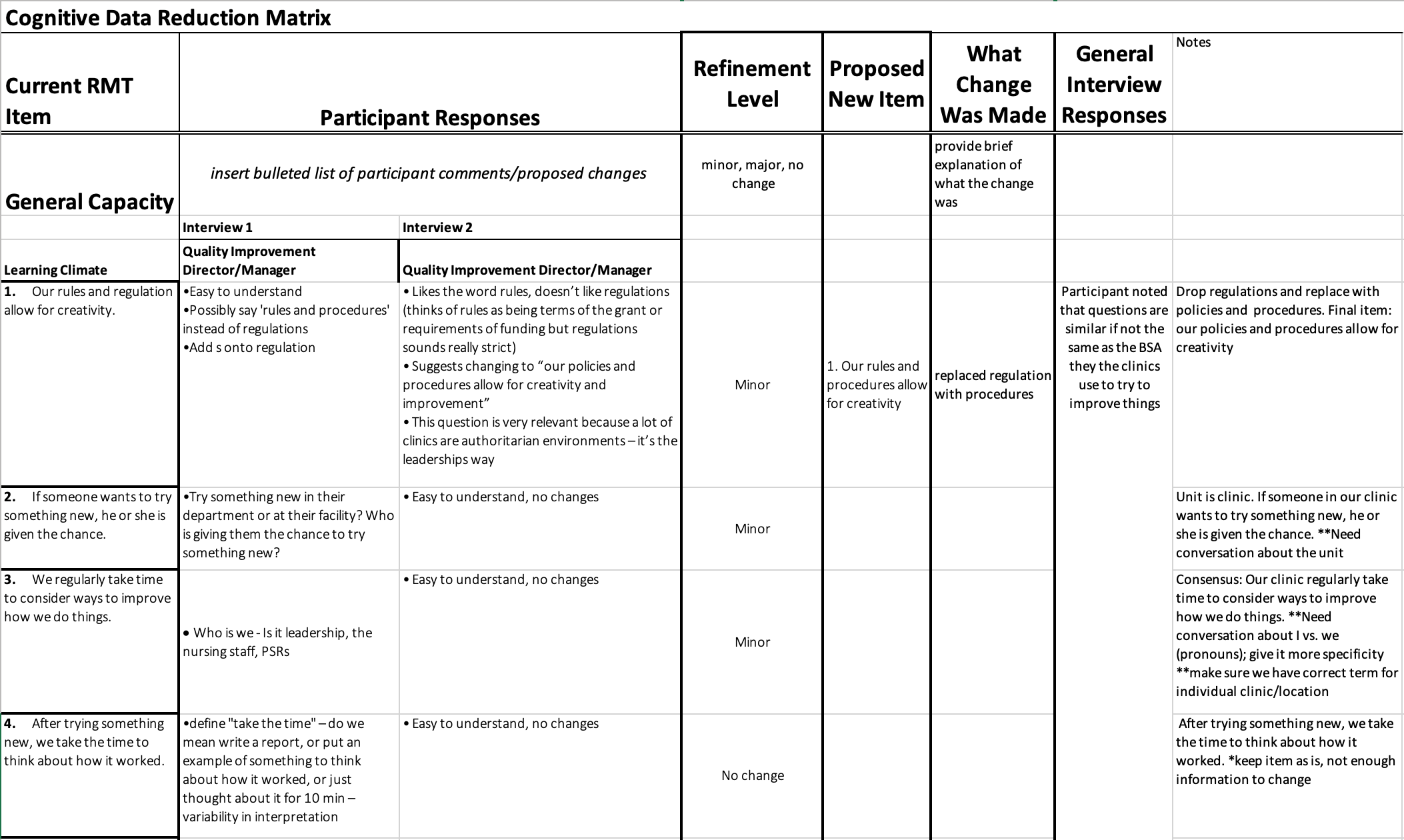

Supplement: Supplementary file 1 — Additional file 1. A Snapshot Example of the Excel Document Used for Data Analysis. [file 12913_2022_9005_MOESM1_ESM.docx]
