# Supplementary material for: Using cognitive interviews to improve a measure of organizational readiness for implementation
Source: BMC Health Serv Res. 2023 Jan 27;23:93. doi: 10.1186/s12913-022-09005-y (PMC9881511; doi:10.1186/s12913-022-09005-y)
Supplement: Supplementary file 2 — Additional file 2. A Snapshot Example of the Full Table Used to Keep Track of Steps Leading to Changes in the Readiness Survey. [file 12913_2022_9005_MOESM2_ESM.docx]

Additional file 2 A Snapshot Example of the Full Table Used to Keep Track of Steps Leading to Changes in the Readiness Survey

**Key**:

* no edits were made; item is unchanged

****** – one or more edits were made to the item

******* – item was flagged for discussion

To make it easier for the reviewers, we underlined words/phrases to highlight the specific changes that were made or things that need further discussion

**Section 1: General Capacity**

| **Learning Climate** | | | | | |
| --- | --- | --- | --- | --- | --- |
| **Current readiness survey Item** | **Interview 1 Response** | **Interview 2 Response** | **Summary of Participants’ Recommendation** | **Readiness Team Thoughts** | **Proposed New Item** |
| 1.    Our rules and regulation allow for creativity. | •Easy to understand  •Possibly say 'rules and procedures' instead of regulations  •Add s onto regulation | • Likes the word rules, doesn’t like regulations (thinks of rules as being terms of the grant or requirements of funding but regulations sounds really strict)  • Suggests changing to “our policies and procedures allow for creativity and improvement”  • This question is very relevant because a lot of clinics are authoritarian environments – it’s the leaderships way | Recommendation: Change ""rules and regulations"" to ""policies and procedures" | Group thought: Agree with and like the participants suggestion | **Our policies and procedures allow for creativity. |
| 2.    If someone wants to try something new, he or she is given the chance. | •Try something new in their department or at their facility? Who is giving them the chance to try something new? | • Easy to understand, no changes | Recommendation: Further specify who we are referring to. | Group thought: we can specify to say clinic or clinic staff  Need conversation about the unit | ***If someone in our clinic wants to try something new, he or she is given the chance. |
| 3.    We regularly take time to consider ways to improve how we do things. | •Who is we - Is it leadership, the nursing staff, PSRs | • Easy to understand, no changes | Recommendation: Specify who takes time to consider ways | Group thought: we can specify by including ""our clinic  -Need conversation about I vs. we (pronouns); give it more specificity  -Make sure we have correct term for individual clinic/location | ***Our clinic regularly takes time to consider ways to improve how we do things. |
